# Repeat hepatectomy for recurrent hepatocellular carcinoma: a local experience and a systematic review

**DOI:** 10.1186/1477-7819-8-55

**Published:** 2010-07-01

**Authors:** Yanming Zhou, Chengjun Sui, Bin Li, Zhengfeng Yin, Yunchang Tan, Jiamei Yang, Zhenyu Liu

**Affiliations:** 1 Department of Hepato-Biliary-Pancreato-Vascular Surgery, the First affiliated Hospital of Xiamen University, Xiamen, China; 2 Department of Special Treatment and Liver transplantation, Eastern Hepatobiliary Surgery Hospital, Second Military Medical University, Shanghai, China; 3 Department of Molecular Oncology, Eastern Hepatobiliary Surgery Hospital, Second Military Medical University, Shanghai, China

## Abstract

**Background:**

This study aimed to assess the efficacy and safety of repeat hepatectomy for recurrent hepatocellular carcinoma (HCC).

**Methods:**

Thirty-seven patients who underwent a curative repeat hepatectomy in our hospital were retrospectively studied. An extensive database literature search was performed to obtain for all relevant studies.

**Results:**

In our series, there were no perioperative deaths during repeat hepatectomy for recurrent HCC. Patients survival after repeat hepatectomy were similar to 429 patients undergoing initial hepatectomy. A computerized search of the Medline and PubMed databases found 29 retrospective studies providing relevant data in 1149 patients were included for appraisal and data extraction. After the repeat hepatectomy, postoperative morbidity ranged from 6.2% to 68.2% with a median per cohort of 23.5 per cent. There were 7 perioperative deaths (0.7 per cent of 993 for whom mortality data were provided). The overall median survival ranged from 21 to 61.5 months, with 1 -, 3 -, and 5-year survival of 69.0% to 100%, 21.0% to 87.0%, and 25.0% to 87.0%, respectively.

**Conclusions:**

Repeat hepatectomy can be performed safely and is associated with long-term survival in a subset of patients with recurrent HCC. However, the findings have to be carefully interpreted due to the lower level of evidence. A randomized controlled study is needed to compare repeat hepatectomy and other modalities for recurrent HCC.

## Background

Hepatocellular carcinoma (HCC) is the fifth most common cancer in the world, responsible for 500,000 deaths globally every year, and its incidence is increasing worldwide because of the dissemination of hepatitis B and C virus infection [1]. With advances in surgical techniques and perioperative care, results of hepatic resection for HCC have greatly improved. Nonetheless, the long-term survival after hepatectomy remains unsatisfactory because of the high incidence of recurrence. Intrahepatic recurrence are the most common and are seen in up to 68-96% of patients [2]. Thus, effective therapeutic strategies of intrahepatic recurrence is critical in prolonging survival after resection of HCC.

Transcatheter arterial chemoembolization (TACE) is most commonly used as a treatment modality for intrahepatic recurrence. However, the role of TACE therapy in the treatment of postoperative recurrence is pessimistic, with 5-year survival rates only range from 0% to 27%, even with repeated TACE treatment [3-5]. Hepatic resection is the only therapy that is potentially curative for liver tumors, and offers patients a chance of long-term survival. Currently, many treatment centers advocate the repeat hepatectomy is the first choice of treatment for recurrent HCC and have claimed that it is safe and that it has similar survival results to initial hepatectomy [6]. However, due to the limited numbers of patients who undergo resection at a single institute, a thorough assessment of the outcome of repeat hepatectomy for recurrent HCC has not been reported.

In this study, based on literature review and retrospective results from our institution, we assessed the efficacy and safety of repeat hepatectomy for recurrent HCC.

## Methods

### The author's experience

#### A) Patients

From December 1999 and June 2005, 462 patients with HCC underwent initial hepatic resection with curative intent at the Department of Special Treatment and Liver transplantation in Eastern Hepatobiliary Surgery Hospital, Second Military Medical University, Shanghai, China. Curative resection was defined as grossly complete removal of the tumor with a clear microscopic margin. Four patients died within 1 month after operation and 29 patients lost follow-up were excluded from the present study. The remaining 429 patients were followed-up every 1 month by tumor marker (alpha-fetoprotein, AFP) analysis and ultrasound or computed tomography at least every 3 months in the first year after hepatectomy, and then at gradually increasing intervals. Recurrence was identified by new lesions on imaging with appearances typical of HCC or a rising AFP level. When findings on ultrasound or computed tomography were inconclusive, hepatic angiography with infusion of iodized oil was performed.

During a median follow-up period of 25 months, 276 (64.3%) patients developed intrahepatic recurrence, and 37 patients underwent a second hepatectomy (rate of repeat hepatectomy, 13.4%). There were 32 men and 5 women with a median age of 52 years (range, 16 to 81 years) at the time of the second operation. Our selection criteria for repeat resection were the same as those for initial resection: good general condition, favorable Child-Pugh Class (A plus selected Grade B), adequate liver remnant, and the ability to technically resect all tumor with curative intent. Treatments for unresectable intrahepatic recurrence were TACE (n = 126), local thermal and chemical ablation (n = 45), and only conservative management (n = 68).

#### B) Operative procedures

Surgery was performed through a right or left subcostal incision or bilateral subcostal incision. After an exploratory laparotomy, the liver was fully mobilized from all its peritoneal attachments. The liver was then assessed with intraoperative ultrasound to assess the extent of local disease, and to detect any extrahepatic metastases or peritoneal seedings. A Pringle maneuver was carried out for controlling the portal triad, with a clamp/unclamp time of 15 min/5 min, during hepatic parenchymal transection if necessary. Transection of the liver was achieved using the Kelly clamp crushing technique. The vascular and biliary radicals were ligated and divided intrahepatically. Fibrin glue was applied to the raw surface of the liver.

Nomenclature for the extent of hepatic resection follows the Brisbane 2000 Guidelines for Liver Anatomy and Resection [7]. Major resection were defined as a resection of 3 or more segments, whereas minor resection were defined as a resection of 2 or fewer segments according to the Couinaud classification. Perioperative mortality was defined as any death either within 30 days of surgery or occurring in the hospital.

#### C) Statistical analysis

Categorical variables were compared by using the Chi-square test or the Fisher exact test as appropriate. Continuous data were expressed as the mean ± standard deviation and compared by one-way ANOVA. Mann-Whitney U test was used to evaluate differences between groups. Overall survival rates were estimated with the Kaplan-Meier method and compared by log-rank test. The Cox proportional hazards model was used for multivariate analysis of prognostic factors. All statistical analyses were performed using SPSS for Windows (version 11.0; SPSS Institute, Chicago, IL, USA). *P *< 0.05 was considered statistically significant.

### Systematic review

#### A) Literature search

Electronic literature searches were performed to identify all published peer-review medical articles on repeat hepatectomy for recurrent HCC. Medline and PubMed databases were searched from the time of inception to November 2009. The following Mesh search headings were used: "recurrent hepatocellular carcinoma," "repeat hepatectomy," "repeat hepatic resection," and "second hepatectomy." Reference lists of all retrieved articles were manual searched for additional studies.

#### B) Selection criteria

For inclusion in review, studies that reported at least 10 patients and that used repeat hepatectomy for recurrent HCC with a curative intent were retrieved. Studies were classified into 4 levels of evidence as follows: level 1, randomized controlled trials; level 2, controlled clinical trials; level 3, observational studies with matched control groups; and level 4, observational observational case series. Letters, reviews, abstracts, editorials, expert opinions, non-English language papers and animal studies were excluded. Studies that included other liver cancer diagnoses were excluded. In the case of multiple publication of a given cohort of patients, the first published article was included in our analysis. However, if a more recent publication corroborated the results of a larger cohort, longer follow-up, or both, we included this more recent publication.

#### C) Data extraction and critical appraisal

Data extraction was performed independently by two authors (Y.M.Z. and B.L., respectively) using predefined criteria. The two investigators independently reviewed all the retrieved studies that met the inclusion and exclusion criteria. Discrepancies between the two reviewers were resolved by discussion and consensus. Each included study was appraised for its level of evidence. The two reviewers extracted data on the following categories: (1) number of patients undergoing surgery for recurrent HCC; (2) resectability rate; (3) postoperative morbidity and mortality; (4) overall survival; (5) prognostic factors. A meta-analysis was not possible because none of the studies were randomized trials. All relevant text, tables and figures were reviewed for data extraction. Data are presented as median (range) unless otherwise stated.

## Results

### The author's experience

Table 1 shows a comparison of the clinicopathological features, operative procedures and perioperative outcomes among the 462 patients who underwent initial hepatectomy and the 37 patients who underwent repeat resection. The initial resection group had larger tumor size and higher aminotransferase level. There were no differences between initial and repeat hepatectomy with respect to Child-Pugh classifications, serum AFP level, total bilirubin level, tumors number and location, tumor capsule formation, vascular invasion, Edmondson-Steiner grade.

**Table 1:** Comparison of clinicopathological features, operative procedures and perioperative outcomes between the initial hepatectomy group and the repeat resection group

Variables	Initial Hx (n = 462)	Repeat Hx (n = 37)	*p *value
Median age at operation (years)	49.0	52.0	NS
Gender (male/female)	419/43	32/5	NS
HBV-positive	451 (97.6)	37 (100)	NS
Child-Pugh class A	424 (91.8)	37 (100)	NS
Serum AFP > 100 ng/mL	212 (45.8)	13 (35.1)	NS
Total bilirubin (umol/L)	14.5 ± 8.9	16.4 ± 8.3	NS
Serum albumin (g/L)	41.8 ± 3.6	41.2 ± 3.7	NS
Serum ALT (IU/L)	56.3 ± 51.8	36.7 ± 11.4	0.028
Serum AST (IU/L)	53.4 ± 52.7	33.0 ± 10.1	0.007
Tumor size in diameter (cm)	7.3 ± 4.8	3.3 ± 1.9	<0.001
Tumor location			NS
Right lobe	326 (70.5)	23 (62.1)	
Left lobe	134 (29.0)	14 (37.8)	
Both lobe	12 (2.5)	0	
Cirrhotic liver	238 (51.6)	20 (54)	NS
Solitary tumor	374 (80.9)	28 (75.6)	NS
Tumor capsule formation	291 (62.9)	26 (70.2)	NS
Vascular invasion	272 (58.2)	19 (51.3)	NS
Edmonson-Steiner grade			NS
G1-G2	31 (6.7)	4 (10.8)	
G3-G4	431 (93.3)	33 (89.2)	
Extent of hepatectomy			0.018
Major resection	133 (28.7)	4 (10.8)	
Minor resection	329 (56.8)	33 (89.2)	
Combined organ resection	114 (24.6)	0	0.001
Operation time (min)	228.6 ± 68.4	211.8 ± 67.0	NS
Clamping time (min)	18.5 ± 8.2	16.7 ± 10.4	NS
Blood loss (mL)	690.0 ± 762.8	364.2 ± 293.4	0.002
Need for blood transfusion	64 (13.8)	6 (16.2)	NS
Postoperative complication	147 (31.8)	9 (24.3)	NS
Mortality	4 (0.8)	0	NS
Postoperative hospital stay (days)	10.6 ± 3.2	11.5 ± 3.1	NS

Hx: hepatectomy. HBV: hepatitis B virus. AFP: alpha-fetoprotein.

ALT: alanine aminotransferase. AST: aspartate aminotransferase.

Major resections were performed more frequently in initial hepatectomy. Combined organ resection was common in initial resection (24.6%). Similarly, initial resection group had more intraoperative blood loss. However, there were no differences between two groups in terms of operating time, clumping time, transfusion requirement, perioperative morbidity and mortality.

The overall 1-year, 3-year, and 5-year survival rates after initial hepatectomy in the whole group of 429 patients were 91.2%, 69.4%, and 42.5%, respectively, these were similar to 37 patients after repeat hepatectomy (94.6%, 70.3% and 43.7%, respectively).

**Figure 1:**
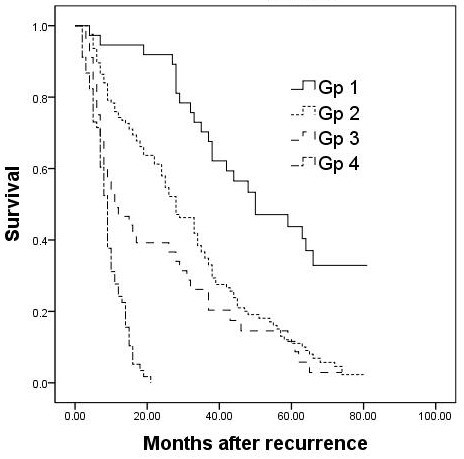
**Overall survival from the time recurrence of patients treated with repeat resection (Gp 1), TACE (Gp 2), local ablation (Gp 3), and conservative management (Gp 4)**.

Figure 1 shows the comparison of survival rates after HCC recurrence according to the types of treatment. The survival rate of patients who had repeat hepatectomy was significantly better than the rates of patients who had non-surgical treatment. The 1-year, 3-year, and 5-year survival rates of patients with TACE were 74.3%, 33.3%, and 11.1%, respectively. Patients who underwent local ablation had 1-year, 3-year, and 5-year survival rates of 46.6%, 20.3%, and 8.8%, respectively. For patients treated with conservative management after recurrence had survival rates of 24.2%, 0%, and 0%, respectively.

Univariate analysis revealed that vascular invasion, multiple recurrent tumors, and a recurrence-free interval of ≤1 year were adverse prognostic factors for survival after repeat hepatectomy (Table 2).

**Table 2:** Prognostic factors for overall survival after repeat hepatectomy according to univariate analysis

Variables	No. of patients	Median survival (months)	*p *value
Age (years)			NS
>60	7	38	
≤60	30	63	
Gender			NS
Male	32	50	
Female	5	65	
Serum AFP (ng/mL)			NS
>100	13	50	
≤100	24	59	
Serum total bilirubin (umol/L)			NS
>20	8	35	
≤20	29	63	
Serum ALT (IU/L)			NS
>40	12	37	
≤40	25	59	
Serum AST (IU/L)			NS
>40	7	48	
≤40	30	59	
Background liver tissue			NS
Non-cirrhotic	17	64	
Cirrhotic	20	44	
Tumors capsule formation			NS
Absent	11	48	
Present	26	64	
Tumors size (cm)			NS
>3	15	50	
≤3	22	59	
Number of tumors			0.040
Solitary	28	64	
Multiple	9	37	
Vascular invasion			0.004
Absent	18	68	
Present	19	38	
Edmonson-Steiner grade			NS
G1-G2	4	50	
G3-G4	33	59	
Recurrence-free interval (year)			<0.001
>1	29	64	
≤1	8	29	

AFP: alpha-fetoprotein. ALT: alanine aminotransferase. AST: aspartate aminotransferase.

Multivariate analysis indicated that the recurrence-free interval of ≤1 year (risk ratio = 2.665, 95% confidence interval = 0.964-7.364, *P *= 0.05) was the only independent prognostic factor for overall survival after repeat hepatectomy.

### Literature search

#### A) Quantity and quality of evidence

This electronic search resulted in the identification of 256 publications. On initial evaluation of these abstracts, 36 studies remained. Manual review of the citation lists identified a further 4 studies. A total of 40 potentially relevant publications were retrieved for further evaluation. Of these, 6 were excluded for the following reasons: 1 study evaluating the impact of obesity on the surgical outcome following repeat hepatic resection patients with recurrent HCC, 2 studies lacks information of survival, 3 were earlier publications from the same treatment center. Another 5 were excluded because the number of patients in each study was fewer than 10. Finally, 29 studies matched the selection criteria and were therefore included. All studies were retrospective in design and their size ranged from 11 to 149 patients. Of these, 28 studies were observational cases series with no control groups and were classified as level-4 evidence [5,8-34], 1 study compared percutaneous radiofrequency ablation versus repeat hepatectomy was classified as level-3 evidence [35].

#### B) Selection criteria for repeat hepatectomy

So far, no consensus has been reached concerning the standard selection criteria for repeat hepatic resection. Generally, patients who had a good performance status and a liver functional reserve, if oncologically radical operation was possible, the patients were selected for hepatectomy [11-33,35].

#### C) Characteristics of the study population

Characteristics of the 29 eligible studies are listed in Table 3. These papers described 1149 patients underwent repeat hepatectomy for recurrent HCC. The rate of repeat hepatectomy ranged from 8.7% to 44.0% (median = 22.8%). The mean age in 17 studies providing data on age ranged from 45.0-66.9 years (median = 59.5). Male: female ratio in the pooled data was 4.2: 1. Median/mean (range) recurrent intervals between the initial and repeat hepatectomy ranged from 6 to 31 (median = 22.4) months. 37.5%-83.3% of patients had solitary intrahepatic recurrence (median = 64.2%).

**Table 3:** Characteristics of the included studies

First author	Year	Intrahepatic recurrences rate (%)	Median recurrent Intervals (months)	Resectability rate (%)	No. of repeat hepatectomy	Male/ female	Mean age (years)	Liver cirrhosis (%)	Solitary recurrent HCC (%)	Maxium tumor size (<3 cm) (%)
Lange [8]	1989	--	9	--	11	9/2	45	36	54.5	27
Nakajima [9]	1993	36.8	--	28.5	14	--	--	50	64.2	64.2
Matsuda [10]	1993	40	18	44	16	13/3	63.3	100	--	--
Wu [11]	1993	--	--	--	72	66/6	45.8	63.9	--	--
Zhou [12]	1993	48.2	--	35	65	--	--	--	--	--
Kakazu [13]	1993	78	26*	11	24	19/5	59.5	--	62.5	--
Suenaga [14]	1994	61.2	33.3*	24.3	18	13/5	56.9	88.8	72.2	67
Lee [15]	1995	40.8	18	31.2	25	17/8	55.8	76	60	32
Nagasue [16]	1996	--	21*	30	50	36/14	59	76	70	--
Shuto [17]	1996	57		16	31	27/4	60	94	64.5	--
Hu [18]	1996	--	23	--	59	46/13	52	--	66.1	--
Shimada [19]	1998	--	--	--	41	33/8	--	56	65.8	70.7
Arii [20]	1998	--	--	12.6	22	16/6	60	68.2	63.6	--
Farges [21]	1998	67	--	16.8	15	--	--	--	--	--
Poon [5]	1999	43	6	10	11	--	--	--	--	--
Nakajima [22]	2001	60.6	31	21	12	10/2	62.3	41.6	83.3	--
Sugimachi [23]	2001	63.3	--	26	78	--	--	--	--	--
Tanabe [24]	2001	56.9	--	19.6	21	--	--	--	76.1	--
Shen [25]	2002	34.6	--	22.4	20	--	--	--	--	--
Minagawa [26]	2003	54.7	--	31	67	56/11	62.7	68.6	55.2	76.1
Chen [27]	2004	56.5	9.0	11.8	34	--	--	--	--	--
Sun [28]	2005	--	26.8	--	57	52/5	52.3	85.9	82.4	--
Kobayashi [29]	2006	55.2	--	29	60	--	--	--	--	--
Tralhão [30]	2007	40	27	21	16	14/2	61.3	93.7	43.7	--
Itamoto [31]	2007	58	22.4	25	84	64/20	66	67	63	63
Wu [32]	2009	54.4	26.3	23.2	149	114/35	59ï¿½0	--	79.1	--
Kawano [33]	2009	67.4	12	8.8	13	--	--	--	--	--
Nagano [34]	2009	45.5		22.8	24	20/4	66.9	--	37.5	--
Liang [35]	2008	--		--	44	39/5	48.8	--	77.2	59.0

* mean

#### D) Operative strategy

At the time of repeat recection, the proportion of patients who underwent minor resection ranged from 71.4% to 100% (median = 95.5%). The median/mean operating time ranged from 136 to 365 (median = 267) min. The median/mean estimated blood loss ranged from 211 to 1980 (median = 603) ml (Table 4).

**Table 4:** Summary of outcomes reported in the included studies

Variable	No. of studies with available information	Median (range)
Intrahepatic recurrences rate after Initial Hx (%)	21^5, 9, 10, 12-15, 17, 21-27, 29-34^	55.2 (34.6-78)
Median/mean recurrent intervals (months) after initial Hx	15^5, 8, 10, 13-16, 18, 22, 27, 28, 30-33^	22.4 (6-33.3)
Resectability rate (%)	23^5, 9, 10, 12-17, 20-27, 29-34^	22.8 (8.7-44)
No. of repeat Hx	29^5, 9-35^	1149*
Mean age (years)	17^8, 10, 11, 13-17, 20, 22, 26, 28, 30-32, 34, 35^	59.5 (45-66.9)
Male (%)	19^8, 10, 11, 13-19, 22, 26, 28, 30-32, 34, 35^	81.2 (68-91.6)
Liver cirrhosis (%)	15^8-11, 14-17, 19, 20, 22, 26, 28, 30, 31^	68.6 (36-100)
Solitary recurrent HCC (%)	19^8, 9, 13-20, 22, 24, 26, 28, 30-32,34, 35^	64.2 (37.5-83.3)
Maxium tumor size <3 cm (%)	8^8, 9, 14, 15, 19, 26, 31, 35^	63.6 (27-76.1)
Minor resection (%)	18^8-18, 22, 26, 30- 32, 34^	95.5 (71.4-100)
Median/mean operating time (min)	9^13, 15, 16, 19, 22, 26, 30, 32, 34^	267(136-365)
Median/mean blood loss (mL)	13^13-16, 19, 22, 26, 28-30, 32, 34, 35^	603 (211-1980)
Morbidity (%)	12^10, 13, 15, 16, 18, 22, 28-32, 35^	23.5 (6.2-68.2)
Mortality (%)	24^5, 8-10, 12-23, 26, 28-32, 34, 35^	0 (0-8.0)
Overall survival after recurrence		
Median time (months)	8^8, 10, 14, 15, 18, 22, 28, 33^	27.1 (21-61.5)
1-year (%)	25^5, 8-22, 25-31,34, 35^	91.0(69-100)
3-year (%)	26^5, 8-24, 26-31, 34, 35^	63.4 (21.0-87.0)
5-year (%)	22^5, 9, 11-14, 16, 17, 19, 21, 23, 24, 26-35^	48.5(25.0-87.0)

Hx: hepatectomy. HCC: hepatocellular carcinoma. * total

#### E) Morbidity and Mortality

After the repeat hepatectomy, data were available on postoperative complication rate for recurrent HCC in 12 studies covering 596 patients, with a median (range) morbidity of 23.5% (6.2-68.2%). A total of 7 deaths were reported in 24 studies covering 993 patients, giving a mean mortality rate of 0.7 per cent. The reported mortality rate in these studies ranged from 0 to 8.0 per cent (Table 4).

#### F) Survival

The overall median survival since the repeat hepatectomy ranged from 21 to 61.5 months, with 1-, 3-, and 5-year survival of 69.0% to 100%, 21.0% to 87.0%, and 25.0% to 87.0%, respectively (Table 4).

#### G) Significant prognostic factors for survival

A few studies have identified the independent poor prognostic factors after a repeat hepatic resection. Factors related to initial hepatectomy included the following: portal vein invasion [19,34], multiple lesion [26], and short recurrence-free interval between initial and repeat hepatectomy (<1 year [26,35], or <1.5 year [34]). Factors related to repeat hepatectomy included the following: female gender, younger age, tumor grade [18], microscopic vascular invasion [31], recurrent tumors >3 cm, and serum albumin level <35 g/L [35].

## Discussion

The postoperative recurrence of HCC remains the major cause of death and the main obstacle to long-term survival. The remnant liver is the primary site of tumor recurrence, the recurrence rate is 36.8-78% in current systemic review. Although various therapeutic modalities have been used for the treatment of recurrent HCC, hepatic resection is the only therapy that is potentially curative for liver tumors, and offers patients a chance of long-term survival. However, repeat hepatectomy is considered unsuitable for majority of patients with intrahepatic recurrence. The rate of repeat hepatectomy for HCC recurrence ranged from 7% to 30% in the present systematic review (the figure in our current study was 13.4%). The main reason is the low rate of resectability in patients with intrahepatic recurrence because of the multifocality, location of the tumor, or degree of cirrhosis [33].

Repeat hepatectomy is more technically challenging than initial resection because of impaired liver function due to the progression of hepatitis, the presence of adhesion, and modifications in the anatomy by the previous operation. However, our study and previous reports compared the perioperative outcomes after initial and repeat hepatectomy and did not find any statistically significant. The overall perioperative morbidity rate ranged from 6.2% to 68.2% (24.3% in our series). These complications were easily managed with conservative management. Although data on postoperative death were provided in only 993 of 1149 patients, a mortality rate of 0.7 per cent is very low. Furthermore, repeat hepatectomy can achieve a long-term survival for patients with recurrent HCC. The overall median survival since the repeat hepatectomy ranged from 23 to 56 months, with 5-year survival of 25% to 87%, and the figure was 43.7% in our series. Moreover, several studies showed that there was no marked difference in survival after the initial and repeat hepatectomy [5,11,14,18,19,26,29,32,34]. These data suggest that repeat hepatectomy is a safely and effective therapy for intrahepatic recurrence.

Predictably, nonsurgical treatment continues to be a factor associated with poor survival of patients with recurrent HCC [5,24,27]. The survival outcome of repeat hepatectomy is considerably better than that of nonsurgical or conservative treatment [5,9,10,12,14-17,21,23-25,27,30,32]. It should be noted that the favourable results of repeat hepatectomy might partly be due to a high selection of patients with a well preserved liver function and limited intrahepatic tumor spread. Patients who did not undergo repeat hepatectomy may have had poorer liver functional reserve and/or too advanced recurrent tumor [32]. The clinicopathological backgrounds of the patients in the different treatment groups were quite different, so comparisons among the various treatments would be of limited value.

Postoperative HCC recurrence is thought to take place in two ways, intrahepatic metastasis (IM) through the portal vein in the residual liver and metachronous, multicentric hepatocarcinogenesis based on chronic hepatitis [36]. Generally, the two kinds of recurrence can roughly be distinguished according to the interval after hepatectomy. The early recurrences (≤1 year) may arise mainly from IM, whereas most of the late recurrences (>1 year) are probably multicentric in origin [36]. Early recurrence have been found to be a significant prognostic factor after repeat hepatectomy in two reports [26,35], and our study confirmed the same findings. These data suggested that patients with late recurrences may be more favorable candidates for repeat hepatectomy.

## Conclusions

Repeat hepatectomy can be performed safely and is associated with long-term survival in a subset of patients with recurrent HCC. Although promising, it must also be emphasized that all current available studies are low level evidence. Thus, randomized controlled study is needed to compare repeat hepatectomy and other modalities for recurrent HCC.

## Competing interests

The authors declare that they have no competing interests.

## Authors' contributions

YMZ participated in the design and coordination of the study, carried out the critical appraisal of studies and wrote the manuscript. CJS, BL, ZYL, and ZFY developed the literature search, carried out the extraction of data, assisted in the critical appraisal of included studies and assisted in writing up. ZFY and YCT carried out the statistical analysis of studies. JMY interpreted data, corrected and approve the manuscript. All authors read and approved the final manuscript.
